# Unsupervised pattern and outlier detection for pedestrian trajectories using diffusion maps

**DOI:** 10.1016/j.physa.2023.129449

**Published:** 2024-01

**Authors:** Fanqi Zeng, Nikolai Bode, Thilo Gross, Martin Homer

**Affiliations:** a School of Engineering Mathematics and Technology, University of Bristol, Bristol, BS8 1TW, UK; b Department of Sociology, University of Oxford, Oxford, OX1 1JD, UK; c Helmholtz Institute for Functional Marine Biodiversity (HIFMB), 26129 Oldenburg, Germany; d Institute for Chemistry and Biology of the Marine Environment, University of Oldenburg, 26129 Oldenburg, Germany; e Alfred Wegener Institute, Helmholtz Center for Marine and Polar Research, 27570 Bremerhaven, Germany

**Keywords:** Diffusion maps, Dimensionality reduction, Trajectory analysis, Pedestrian dynamics, Model validation, Outlier detection

## Abstract

The movement of pedestrian crowds is studied both for real-world applications and to gain fundamental scientific insights into systems of self-driven particles. Trajectory data describes the dynamics of pedestrian crowds at the level of individual movement paths. Analysing such data is a central challenge in pedestrian dynamics research, coupled with increasing data availability this implies a need for efficient methods to identify key features of the captured crowd dynamics. In this paper, we show that diffusion maps, an unsupervised manifold learning method, can be used for this purpose. We show how to build an informative feature space by defining a set of observables from trajectories. We use our diffusion map approach to analyse pedestrian movement on a stadium-shaped track, and during egress from a room, considering hundreds of trajectories for each scenario. We first verify that our diffusion map analysis can recover known leading variables that determine the system dynamics. Then, we show how our analysis facilitates a qualitative comparison of the dynamics inherent in entire data sets, by contrasting experimental with numerically simulated data. Finally, we establish how our approach can be used to automatically detect outliers that show behaviour distinct to others. These results indicate that our work can contribute a computationally efficient and unsupervised approach to analyse pedestrian dynamics without needing much prior knowledge of the data. We suggest this could be useful for automatically monitoring live data, or as an initial step to inform a subsequent analysis.

## 1 Introduction

The movement of pedestrian crowds is an example of collective human behaviour that is directly relevant to both scientific and industrial communities. The development of data recording and analysis techniques, coupled with advances in computer vision and smartphone sensors, has led to a substantial increase in data on pedestrian movements over the last two decades [1]. This wealth of data means employing fast and unsupervised learning methods to search for patterns and outliers in pedestrian data is now a meaningful approach. Unsupervised learning methods – in contrast to supervised methods – do not ask for labelled data to identify patterns in the data. Advantages of such unsupervised data analyses are that they provide a fast way to generate insights into the main drivers of variability in data, can uncover patterns that are difficult to spot by eye, and identify outliers without the need to sift through data manually. They can thus be used to simplify high-dimensional data sets, either to generate insight in its own right, or to inform further analysis.

There are two main approaches for using unsupervised learning methods in pedestrian dynamics research. First, to aid data extraction from sensor recordings, such as videos. Second, to aid data analysis and modelling, mostly via dimensionality reduction. Each of these approaches will be briefly discussed below.

Considering the first approach, as part of developing computer vision techniques, researchers have implemented unsupervised learning methods. For example, Zou et al. [2] proposed an unsupervised framework to conduct tracklet (short stretches of tracked movement paths) clustering in crowded video scenes based on a locally consistent latent Dirichlet allocation model. Their application of the framework to three public surveillance video datasets can infer more discriminative and compact motion patterns, such as the predominant movement directions present in the crowd. Luo et al. [3] used an unsupervised learning approach that compactly encodes the motion dependencies in videos to learn long-term temporal representations for videos across different modalities. Given a few video frames, they aim to predict the sequence of ensuing basic motions that could subsequently be used to classify activities. They examined the method’s effectiveness on multiple data sets by using a sequence of atomic 3D flows as supervision to train the model.

Considering the second approach for the use of unsupervised learning methods, different aspects of pedestrian dynamics have been investigated. Wang et al. [4] used a novel graph neural network-based method to predict pedestrian trajectories in a crowd. Their unsupervised method transferred pedestrians’ spatial coordinates and time information into interactive graphs to make predictions. Marschler et al. [5] employed diffusion maps to study the coarse-grained behaviours of two simulated pedestrian crowds trying to pass through a door from opposite sides. The unsupervised diffusion map method was used to reduce the dimensionality of trajectory data by describing it in terms of new variables that capture the dynamics. Lehmberg et al. [6] use a Koopman model and diffusion map to study empirical and simulated pedestrian traffic data in cities. As in the Marschler et al. [5], diffusion maps were used to reduce the dimensionality of the data. Their results showed the superior predictive performance of the method to a naive baseline model, as well suggesting the usefulness of the diffusion map in data-driven analysis.

Here, we propose to use diffusion maps, an unsupervised manifold learning method [7,8], to directly study aspects of the dynamics of pedestrian systems. In contrast to previous work in pedestrian dynamics, here we apply diffusion maps to explore what can be learned directly from the diffusion map manifolds, making use of the ability of diffusion maps to extract leading variables in a complex system without supervision, a property which has been broadly applied in finding patterns in various complex systems. For example, Barter and Gross [9] applied the diffusion map on census data to find the leading social variables, such as the percentage of the student population, that describe the main variation in census responses across urban areas in UK cities. Diffusion maps have also been used in a similar way across systems, including cortical organisation embedding [10], bacterial metabolic niche space mapping [11,12], wireless localisation [13], and biodiversity estimation [14].

In this paper, we implement diffusion maps on openly available data from two different examples of pedestrian dynamics to demonstrate the capability and novelty of the method. The examples from which the data are drawn vary in terms of the environment; they are referred to here as the closed and open systems. In the closed system, the movement of pedestrians is constrained along a stadium shape and, therefore, approximately one-dimensional [15–17]. In the open system, pedestrians are asked to leave a room through one bottleneck and therefore move in two dimensions with a shared predominant movement direction [18–20]. We use the closed system to demonstrate the principle of our use of the diffusion map and demonstrate that the important quantities known to drive pedestrian dynamics (i.e., density) can be recovered, as would be expected from a functioning unsupervised approach. Using the open system, we demonstrate how the diffusion map can be used to directly investigate pedestrian dynamics by considering two critical problems in pedestrian dynamics research.

First, we compare high-dimensional time series data obtained from experiments and simulations. As recording data on pedestrian crowds is expensive and logistically difficult, e.g. consider the remuneration of many volunteers [21], and because of the potential they offer in terms of testing hypotheses and making predictions, computer simulation models are a popular and successful tool in this research field [22]. However, many of these models produce high-dimensional time series data, such as the movement paths of many pedestrians walking simultaneously. To validate these models, it is necessary to compare such data to similarly high-dimensional experimental or observational data of pedestrians [23–26]. Here, we propose an unsupervised qualitative approach for establishing the overlap between computer simulated and experimental pedestrian trajectory data, using simulations of a commonly referenced social force model [27].

The second problem we investigate relates to the variability inherent in pedestrian crowds. Individual pedestrians have different physical and psychological characteristics, or may find themselves in different environmental or social surroundings [28], all of which can affect how they move, including their body movements, direction, and speed. In the context of pedestrian dynamics, it is of interest to not only identify different classes of individuals [29], but also to identify outliers that are very different to others. Here, we demonstrate how the diffusion map can be used as an unsupervised method to identify individuals that can be described as outliers in terms of their movement in pedestrian systems. Subsequent analysis, after detecting such individuals can then be undertaken to investigate the underlying reasons for their differences. Previous approaches for outlier detection in pedestrian crowds are commonly based on computer vision. For example, Nanda et al. [30] used a two-layer neural network to capture and model pedestrian shapes for outlier detection from a single camera. Mehran et al. [31] built a social force model based on crowd videos to detect and localise abnormal behaviour in the crowd. These approaches presented high accuracy in outlier detection but are computationally expensive as they need to deal with objects’ shapes and even environments, rather than just coordinates, in every video frame. Our work here presents a computationally efficient method to conduct similar outlier detection by only using pedestrian trajectories extracted from crowd videos.

## 2 Methods and data

### 2.1 Experimental and simulated data

**Fig. 1:**
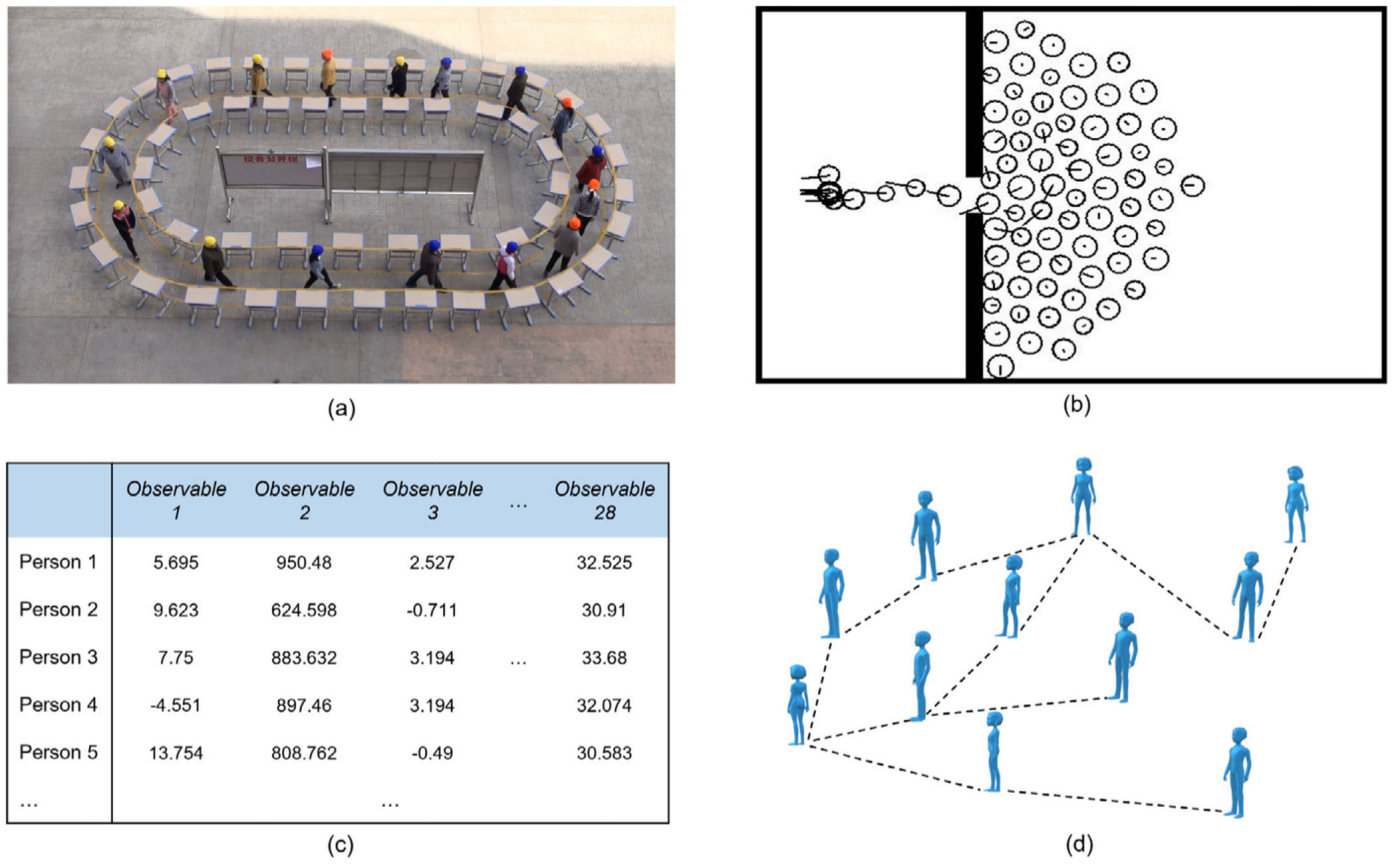
Workflow of diffusion map analysis. (a) Screenshot of the closed experiment system [15], reproduced with permission (screenshot from video material on https://doi.org/10.34735/ped.2015.1; accessed 23/03/2023). (b) Screenshot of an open system simulation. A pedestrian is indicated by a circle with a short straight line showing the movement direction. (c) Illustration of features extracted from the movement of each pedestrian. (d) A network of pedestrians in feature space. Thresholding of similarity scores means that pedestrians are not connected to all other pedestrians. The first step of the diffusion map method is to collect trajectory data, either from experiments or computer simulations. Then, features for individuals based on their movement are computed. Once the features of individuals have been obtained, we can implement the diffusion map and use the information from leading eigenvectors to detect patterns in the data.

In this work, we investigate five different data sets covering two types of pedestrian dynamics. The first data set [15], that we refer to as the closed system, covers the first type of pedestrian dynamics. It comes from 34 experimental runs where pedestrians move in a confined stadium-shaped corridor as shown in Fig. 1(a). In total, 80 young students and 47 older people participated in the experiment. In each experimental run, the number of pedestrians in the ring was varied to achieve different global densities of the crowd. Across runs, the composition of the pedestrian crowd was also changed, including different proportions of older or younger pedestrians. The positions of participants in each run of the experiment were tracked at 25 frames per second. Additional details for the experiment can be found in [16]. Descriptive statistics of this closed experiment data set can be found in Table 1, which reports an overall picture of the size (no. of trajectories), and variations in travel distances and travel time steps (i.e., dimensions of trajectories) of pedestrians in this data set.

The remaining four data sets, that we collectively refer to as the open system, are all for the second type of pedestrian dynamics. All consist of trajectories for individual pedestrians (e.g., time series of locations $\left[\left(x_{t_{1}},y_{t_{1}}\right),\left(x_{t_{2}},y_{t_{2}}\right),\ldots \right])$, as the first data set. They are comprised of one experimental [18] and three computer simulated data sets of crowd egress from a room through one bottleneck (see Fig. 1(b)). The experimental data includes 32 experimental runs with pedestrian numbers ranging from 93 to 127. Experimental runs are performed under three different conditions related to egress competitiveness. A preliminary analysis suggested that our method did not clearly distinguish between runs under different experimental conditions. Hence we will not further investigate these conditions here. Full details of this experiment can be found in [18]. The descriptive statistics of this open experiment data set are presented in Table 1.

The three simulated open system data sets each contain 50 simulation runs of 80 simulated pedestrians motions. We use a derivative of a well-established model for pedestrian dynamics [27] that is often referred to as a Social Force Model to generate the simulated data. Briefly, the model describes the movement of individuals in continuous two-dimensional space by implementing forces acting between simulated pedestrians and the physical environment. These forces are designed to maintain a separation between pedestrians and walls, and separate forces capture friction and other physical effects when these become relevant. Simulated pedestrians seek to exit the room, and their preferred movement direction, including navigating towards and through a bottleneck, is implemented via a floor field. Simulations of this model numerically solve the equations of motion described by the forces using small time steps of 0.05 s, and produce trajectories for all simulated pedestrians. These trajectories are used in our analysis analogously to our use of trajectories from experimental data. For full details of this agent-based model, see [32].

We perform three different types of simulations that differ in terms of the individual characteristics (i.e., shoulder size and movement speed) of simulated pedestrians and the physical environment in simulations. All simulations implement egress from a square room with one bottleneck and a side length of 10 m. Simulated pedestrians are assigned uniformly random starting positions within this room, ensuring that pedestrians do not overlap at the start of simulations. For the first set of simulations (open simulation 1), the width of the bottleneck is set to 90 cm, and for the other two simulations, it is set to 200 cm. In the first and second sets of simulations (open simulations 1 & 2), the shoulder size of pedestrians was drawn randomly from a uniform distribution between 0.2 m and 0.3 m, while their preferred movement speed is 0.6 m/s. All other model parameters were the same for all simulated pedestrians; we used the same parameter values as previously published [32]. For the third set of simulations (open simulation 3), all pedestrians had the same shoulder size of 0.5 m, but their preferred movement speeds were drawn randomly from a uniform distribution between 0.6 m/s and 0.7 m/s. This resulted in three sets of simulations that varied in more and less obvious ways in the resulting dynamics (consider the difference between simulations with different bottleneck widths, which results in a substantial difference in the egress performances, and the difference in the size and speeds of individuals, respectively). As the statistics in Table 1 illustrate, pedestrians in simulation 1 spend on average more time and travel longer than those in simulations 2 and 3. Here, for the simulated data, the time interval between consecutive time steps is 0.05 s; while for the experimental data, the time interval is 0.02 s (i.e., 50 frames per second).

Videos for experiments of the open system are available online [18]. We noticed that in the open experiment, there is a short corridor after pedestrians exit the room. For consistency, we used trajectories inside the room and 0.5 m behind the bottleneck for the simulated and experimental data sets.

### 2.2 Diffusion map procedure

As an unsupervised manifold learning method, diffusion map can be implemented to extract low dimensional manifolds that contain determinant information from high dimensional data [7,8,33]. It can be used for multiple tasks such as data visualisation and clustering in data analysis. The procedure of a diffusion map analysis includes: (i) standardising and then assembling the data set of features; (ii) computing Euclidean distances between data points via a kernel that describes the similarity between samples to construct an affinity matrix; (iii) using this affinity matrix as a weighted adjacency matrix and compute the corresponding Laplacian matrix; (iv) mapping data points in low dimensional space with the spectra of the Laplacian matrix. The driving variables can be identified by the eigenvector corresponding to the smallest non-zero eigenvalue, then the second smallest eigenvalues, and so on. In the original diffusion maps description [8], the eigenvalues are at 1 and go to 0, as the matrix is a stochastic matrix representing the diffusion process. While, it is also possible to consider the generator of the process like the description in the study here.

Notably, the standardisation is not necessary in the original diffusion maps algorithm (as described by [8]). It is mainly required here due to the features are heterogeneous with different scales. Overall, this procedure has only a single parameter *p* related to constructing the affinity matrix and controlling the number of neighbours pedestrians have in feature space. As a result of *p*, the affinity matrix now becomes sparse. Considering the populations in both experimental and simulated groups, we choose *p* = 20 in this work, as discussed in more detail below.

From the trajectories of pedestrians, we compute features and construct an *M* × *N* data matrix ***Φ*** = [*ϕ*_*mn*_] where *M* is the total number of pedestrians from data sets, and *N* is the number of features for an individual pedestrian. Since the trajectories can have varying lengths, this mapping is also necessary as diffusion maps needs a fixed set of features (otherwise the trajectories could also be used as high dimensional data inputs directly). Here, we propose to use 27 features to measure the behaviour of every pedestrian. The details of these features are provided in the next section.

Once we obtained the data matrix ***Φ***, we can standardise it to ensure each observable is of the same scale. To yield a standardised data matrix $\hat{\Phi }$, we compute its component

(1) $${\hat{\phi }}_{mn}=\frac{\phi_{mn}-\mu_{n}}{\sigma_{n}}$$

where *μ*_*n*_ and *σ*_*n*_ are the mean and standard deviation of column *n* of ***Φ*** respectively, which will ensure each observable (column) in $\hat{\Phi }$ has a mean value of zero and a standard deviation of 1. In this work, we consider two different ways to standardise the closed and open system data sets as we want to show the versatility of the diffusion map in these different analyses. For the closed system data sets, we combine them as a single data set and then perform standardisation, since the pedestrians behave similarly (walking along the same closed track) in all runs. For the experimental and simulated data sets in the open system, we standardise every run individually rather than standardise all the combined runs together as the variants of pedestrians’ behaviour are more diverse than in the closed system. Meanwhile, we can check whether the diffusion map can really distinguish different data sources as an unsupervised learning method. The intention for different standardisation methods is that, for the closed system, we demonstrate the method by recovering globally relevant relationships (e.g., based on density). In contrast, for the open system, we investigate if we can classify according to the data source, in which case it makes sense to standardise runs separately.

Each row in $\hat{\Phi }$ can be regarded as a sample in the *N*-dimensional space where entries are coordinates that indicate the corresponding pedestrian’s features. We can then define a *M* × *M* distance matrix **D** where

(2) $$D_{ij}=\sqrt{\underset{n}{\sum }{\left({\hat{\phi }}_{in}-{\hat{\phi }}_{jn}\right)}^{2}}$$

that measures the Euclidean distance of features between pedestrian *i* and *j*.

From the distances between data points, we construct a similarity matrix **C** such that

(3) $$C_{ij}=\{\begin{array}{cc} \Gamma \left(D_{ij}\right) & i\neq j\\ 0 & i=j \end{array}$$

where *Γ* is an affinity kernel. There are various ways to create this kind of kernel, but the selected kernel must be symmetric, positivity preserving, and positive semi-definite [7], such that **C** is symmetric and *C*_*ij*_ ≥ 0. Here we choose *Γ*(*d*) = 1/*d*, meaning that high scores of *C*_*ij*_ will indicate the close affinity of two individuals in the feature space.

In the feature space, large Euclidean distances have negligible influence in defining local similarities, and can thus be disregarded when above certain threshold. So, to reduce redundant information, we threshold the similarity matrix **C** to hold only the high scores between an individual and its closest neighbours while setting all other entries to zero. Here, we choose a heuristic procedure where we only store an entry *C*_*ij*_ if it is among the top *p* highest similarity scores for either individual *i* or *j*. Otherwise, it is set to zero. From a network science perspective, **C** can be regarded as the weighted adjacency matrix of a network with individual pedestrians being nodes and edges linking them to the most similar other pedestrians. The thresholding process described above guarantees each node has at least 20 links in the network, whilst insufficiently similar connections are removed altogether. The benefits of conducting such procedures are that we can obtain a sparse network which is numerically more efficient in computation and avoid the curse of dimensionality. Noteworthily, connections that are actually relevant may be cut off here with the k-nearest neighbours approach (particularly in dense point regions). So, one future direction could be to investigate whether radius-based nearest neighbours approaches to sparsify the affinity matrix are also eligible.

We define the random walk normalised Laplacian matrix **L**, for the network, an *M* × *M* matrix with entries

(4) $$L_{ij}=\;\{\begin{array}{cc} -\frac{C_{ij}}{\sum_{n}C_{nj}} & i\neq j,\\ 1 & i=j. \end{array}$$

The matrix **L** measures a diffusion process among the nodes of the network akin to performing random walks on the network. Notably, we here use the row normalised Laplacian according to the analysis in [14], wherein the authors compared different Laplacians, including those in the original diffusion maps [8].

We can now explore the embedding of the pedestrian traits on low dimensional manifolds by computing the eigenvalues and eigenvectors of **L**. Since Laplacian matrices are positive and semi-definite, they will have real non-negative eigenvalues [34]. Moreover, as the number of zero eigenvalues of **L** is equal to the number of connected components in the network of data points [34], we expect to find exactly one zero eigenvalue, which has a corresponding eigenvector that does not contain any information and is disregarded. If more than one zero eigenvalue exists, the network has become disconnected in the thresholding step, and a less aggressive threshold should be selected.

The smallest positive eigenvalues from **L** are the most informative, as their corresponding eigenvectors span the main directions of the manifolds in the data, i.e., they encode directions of a large variation in the data [9]. Their corresponding eigenvectors allocate the individual pedestrian traits along the main directions of the low dimensional manifold that captures the crucial variations in the data. In the following, we consider the top 3 smallest positive eigenvalues, as in previous work [9,11]. Considering more eigenvalues and corresponding eigenvectors is possible but becomes less informative in terms of the main variability in the data.

### 2.3 Mapping trajectories to features

Before we start implementing the diffusion map, we need to reduce the dimensionality of the data to simplify the analysis, as here, the trajectories can have varying lengths. We propose 27 features that characterise the pedestrian movement dynamics, as shown in Table 2. These features are derived from the trajectories of pedestrians that only consist of positions and velocities at each time step. Based on an individual’s position and velocity, we can identify its nearest neighbour in the run and its second and third nearest neighbours. Then we can calculate the distances and angles for these neighbours. In addition, we calculate the travel distance over 20 time steps and heading direction variances in various time steps for every pedestrian. Here we mainly use the mean values from trajectories for feature computation, which can avoid the impact of different frame rates from different data sets. After obtaining these values at each time step, we can calculate the averages and variances of these features across the whole period for each pedestrian separately, as Table 2 shows. After generating the features data table from the pedestrians’ trajectory table, we can apply the diffusion map approach to the data.

The trajectory data of pedestrian *i* provides its corresponding position vectors **c**_*i*_(*t*) = (*x*_*i*_, *y*_*i*_)_*t*_ and unit direction vectors **v**_*i*_(*t*) = (*dx*_*i*_, *dy*_*i*_)_*t*_ or the whole run. To avoid the mathematical issue of division by zero, here we assume that if pedestrian *j* does not move at time step *s* before they finish the run, they will inherit their previous time step’s velocity, i.e., **v**_*j*_ (*s*) = **v**_*j*_ (*s* − 1). The related distances and angles of pedestrian *i* that we calculate are as follows:

#### Aheadness

We define the *j*th aheadness of individual *i* to its *j*th nearest neighbour at time step *t* as *h*_*ij*_ (*t*) where $h_{ij}(t)=\frac{\left(c_{j}(t)-c_{i}(t)\right)\cdot v_{i}(t)}{\left||v_{i}(t)|\right|}$ which is equal to the length of the (**c**_*j*_ (*t*) − **c**_*i*_ (*t*)) projection onto **v**_*i*_ (*t*), with a minus sign if the direction of the projection is opposite to the direction of **v**_*i*_(*t*). Hence, at time step *t*, the first, second, and third aheadness of individual *i* are *h*_*i*1_(*t*), *h*_*i*2_(*t*), and *h*_*i*3_(*t*), respectively.

#### Leftness

We define the *j*th leftness of individual *i* to its *j*th nearest neighbour at time step *t* as *l*_*ij*_ (*t*) where *l*_*ii*_ (*t*) = $l_{ij}(t)=\frac{\left(c_{j}(t)-c_{i}(t)\right)\cdot v_{i}{\left(t\right)}^{⊥}}{\left||v_{i}(t)|\right|}$, and **v**_*i*_ (*t*)^⊥^ is **v**_*i*_ (*t*) rotated 90° to the left. Hence, at time step *t* the first, second, and third leftness of individual *i* are *l*_*i*1_(*t*), *l*_*i*2_(*t*), and *l*_*i*3_(*t*), respectively.

#### Distance

We define the *j*th distance of individual *i* to its *j*th nearest neighbour at time step *t* as *d*_*ij*_ (*t*) where *d*_*ij*_ (*t*) = ||**c**_*j*_ (*t*) − **c**_*i*_ (*t*)||, which measures the Euclidean distance between these two individuals. Hence, at time step *t*, the first, second, and third distance of individual *i* are *d*_*i*1_(*t*), *d*_*i*2_(*t*), and *d*_*i*3_(*t*), respectively.

#### Adjacent angle

We define the *j*th adjacent angle of individual *i* to its *j*th nearest neighbour at time step *t* as *γ*_*ij*_ (*t*) where $\gamma_{ij}(t)=\arccos\left(\frac{v_{i}(t)\cdot v_{j}(t)}{\left||v_{i}(t)||\;||v_{j}(t)|\right|}\right)$. Hence, at time step *t*, the first, second, and third adjacent angle of individual *i* are *γ*_*i*1_ (*t*), *γ*_*i*2_ (*t*), and *γ*_*i*3_(*t*), respectively.

#### Angle difference

We define the *k*-steps angle difference of individual *i* per *k* time steps as $\alpha_{i}^{k}(t)$ where $\alpha_{i}^{k}(t)=\arccos\left(\frac{v_{i}(t)\cdot v_{i}(t-k)}{\left||v_{i}(t)||\;||v_{i}(t-k)|\right|}\right)$, which measures the angle difference that individual *i* has in every *k* steps.

#### Travel distance

We define the *k*-steps distance of individual *i* per *k* time steps as $s_{i}^{k}(t)$ where $s_{i}^{k}(t)=\sum_{\tau =0}^{k-1}\left||c_{i}(t-\tau )-c_{i}(t-\tau -1\right)||$, which measures the Euclidean distance that individual *i* travels in *k* steps.

## 3 Results

### 3.1 Closed pedestrian system

We first apply our diffusion map approach to the closed pedestrian system data set to demonstrate the concept and to confirm it captures the relevant driving variable in the system.

**Fig. 2:**
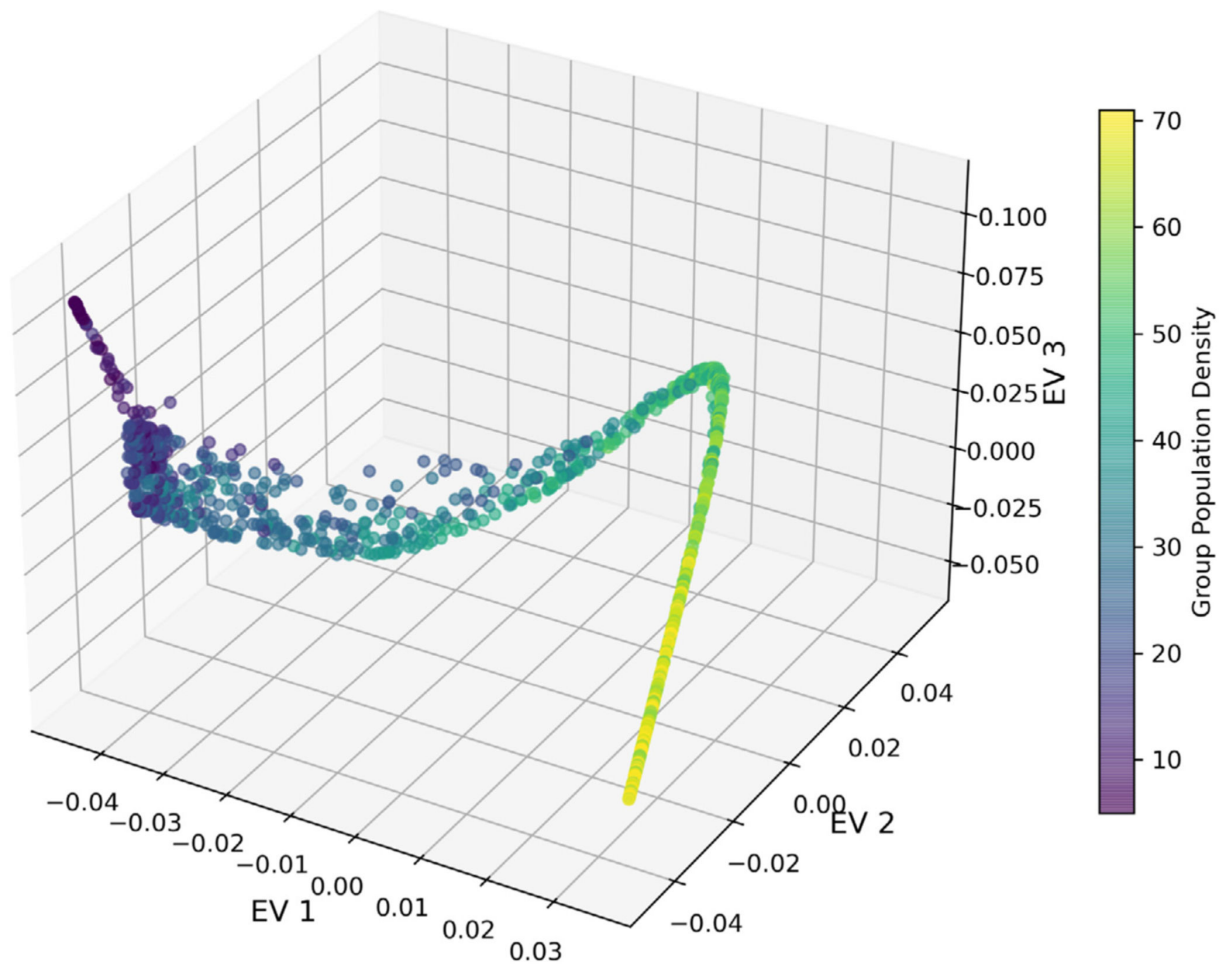
Mapping group density of experiments in the closed system. The axes represent the entries in the top three eigenvectors (EVs) from our diffusion map analysis. Each dot relates to a pedestrian from the closed system data set. Dots are coloured according to the population density of the pedestrian’s group. The colour bar indicates the population size of individual pedestrian’s group. The numbers for the EVs on the three axes are from the entries of the eigenvectors, respectively, and thus dimensionless. Pedestrians from all available experimental runs in the closed data set are included in this analysis. Along the manifold, a shift of colours begins from deep purple (upper left, low-density pedestrians) to yellow (lower right, high-density pedestrians). The three EVs here point this pattern out.

We consider the top three leading eigenvectors (EVs) from the closed system and project all the pedestrians into the corresponding eigenspace, as shown in Fig. 2. The data points in the figure represent the position of individual pedestrians in the eigenspace obtained from our analysis. The figure shows a one-dimensional sub-structure in the three-dimensional eigenspace, also known as manifold, which indicates a pattern characterising the high-dimensional data.

Previous work has shown that the main driving variable in the closed system, which is also varied experimentally, is the pedestrian density [16]. We use the group population *N* in experimental runs as a proxy for group density since the domain is fixed across experiments, and colouring pedestrians in eigenspace according to the group density shows that the density changes systematically from low to high values along the manifold (Fig. 2). Thus, the manifold obtained from our analysis can be directly related to the density, demonstrating that the pattern we find captures the key driving variable in the system. While this variable, the density, is known here, this analysis suggests that our approach can be used to detect meaningful patterns in high dimensional pedestrian data.

### 3.2 Open pedestrian system: Distinguishing datasets

Recall that for the open system, we address two research questions. First, we use the diffusion map approach to qualitatively investigate the overlap between data sets, specifically between experimental and computer simulated data. Second, we use our approach for outlier detection in high-dimensional pedestrian trajectory data. The first question is covered in this section, and the second question is covered in the following section.

We applied our diffusion map analysis to the four open system data sets: one experimental data set, and three simulated data sets with different environment and pedestrian characteristics. It should be clear that the experiment with human participants and the computer simulations differ in many ways. For example, the body shape of pedestrians in simulations is assumed to be circular, when in reality, it is more ellipsoid, and pedestrians can rotate it to efficiently use available space, which is especially important in crowded egress situations [35]. Similarly, the biomechanics of walking are not considered in the model (meaning the simulated trajectories lack features arising from pedestrians shifting their weight from one foot to the other), the choice of model parameters is ad hoc and based on the literature only, when ideally, the model should be calibrated on data (e.g., [23]), and more generally the fundamental assumptions of behaviours implemented in the social force model [27] can be questioned (e.g., [36,37]). Nevertheless, it is interesting to consider the extent to which the dynamics observed in the different data sets overlap. Our diffusion map analysis provides an unsupervised approach for this.

**Fig. 3:**
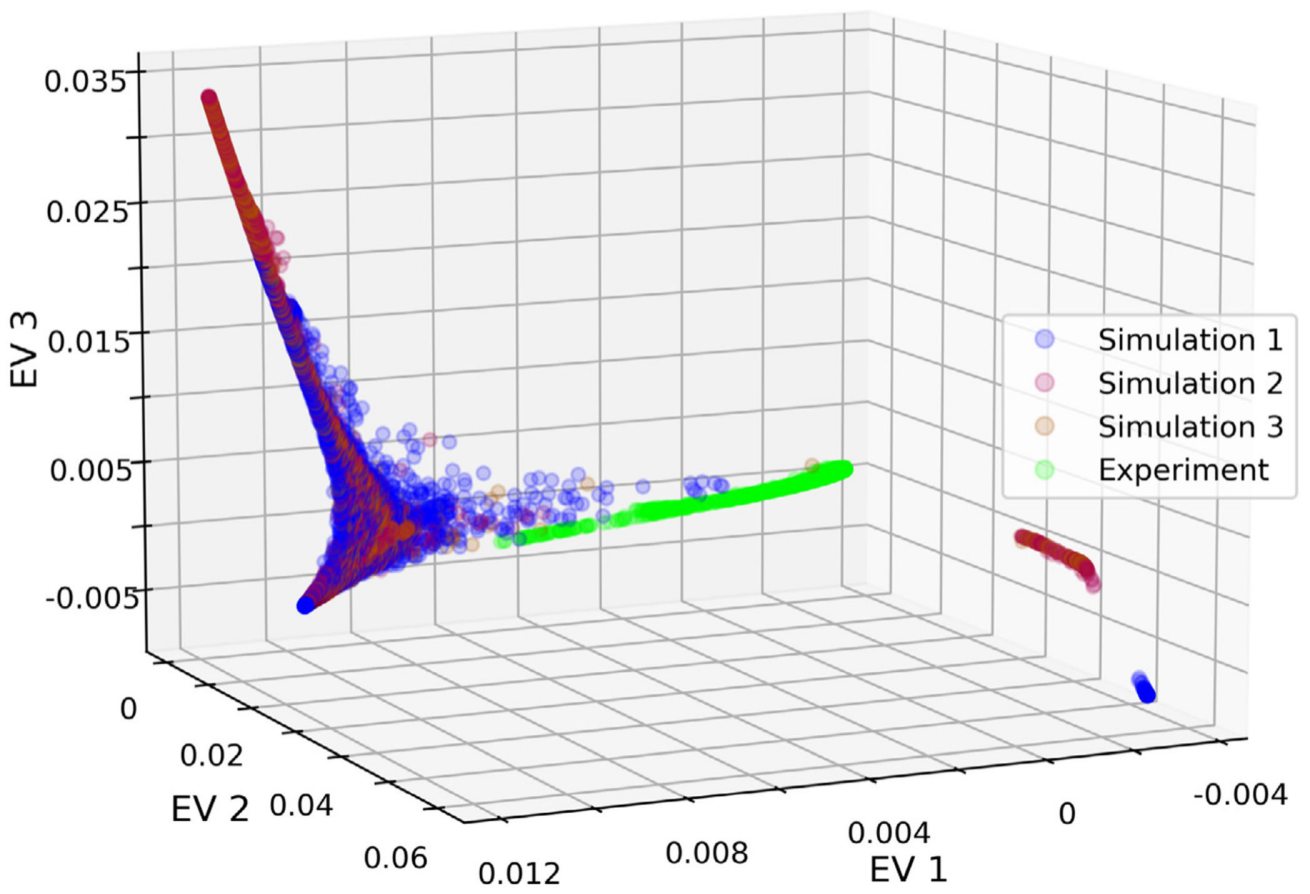
Diffusion map analysis of the four open system pedestrian data sets. Each datum represents an individual pedestrian in the three-dimensional eigenspace defined by the three leading eigenvectors. Colours indicate the data set. The numbers for the EVs on the three axes are from the entries of the eigenvectors, respectively, and thus dimensionless.

Fig. 3 shows the manifold obtained in the eigenspace for the three leading eigenvectors when applying our diffusion map analysis to all four data sets (recall that the standardisation of data is per experimental run). As before, each point also represents an individual pedestrian. We can see that the three manifolds from simulated data overlap substantially with each other. This is perhaps unsurprising, though reassuring, as they have very similar mechanisms except for pedestrian characteristics and simulated environment variations. Nevertheless, the simulation manifolds are not identical, indicating differences among the data sets that could be investigated further by considering additional eigenvectors. In contrast, the manifold for the experimental data is close to a part of the three simulation manifolds, but the overlap is limited. This suggests that while there is some similarity between model simulations and data, our analysis detects underlying differences. The reasons for this are beyond the scope of this work. Possible explanations would be incorrect parameter selection in the models, or some more fundamental disparity between model and experiment.

### 3.3 Open pedestrian system: Outlier detection

In this section, we demonstrate the usefulness of our diffusion map analysis for outlier detection in high-dimensional pedestrian dynamics data.

We conduct the outlier detection on the collection of all four data sets from the open system. We define outliers as the data points with extreme values in the top eigenvectors, for example, the first maximum and minimum entries in the first or second eigenvectors. These extreme data points can usually be found at the end or boundaries of the manifold in the eigenspace, as shown in Fig. 3. After detecting outliers in this way, we can investigate their corresponding trajectories further, for example, in comparison to other trajectories in the data set.

**Fig. 4:**
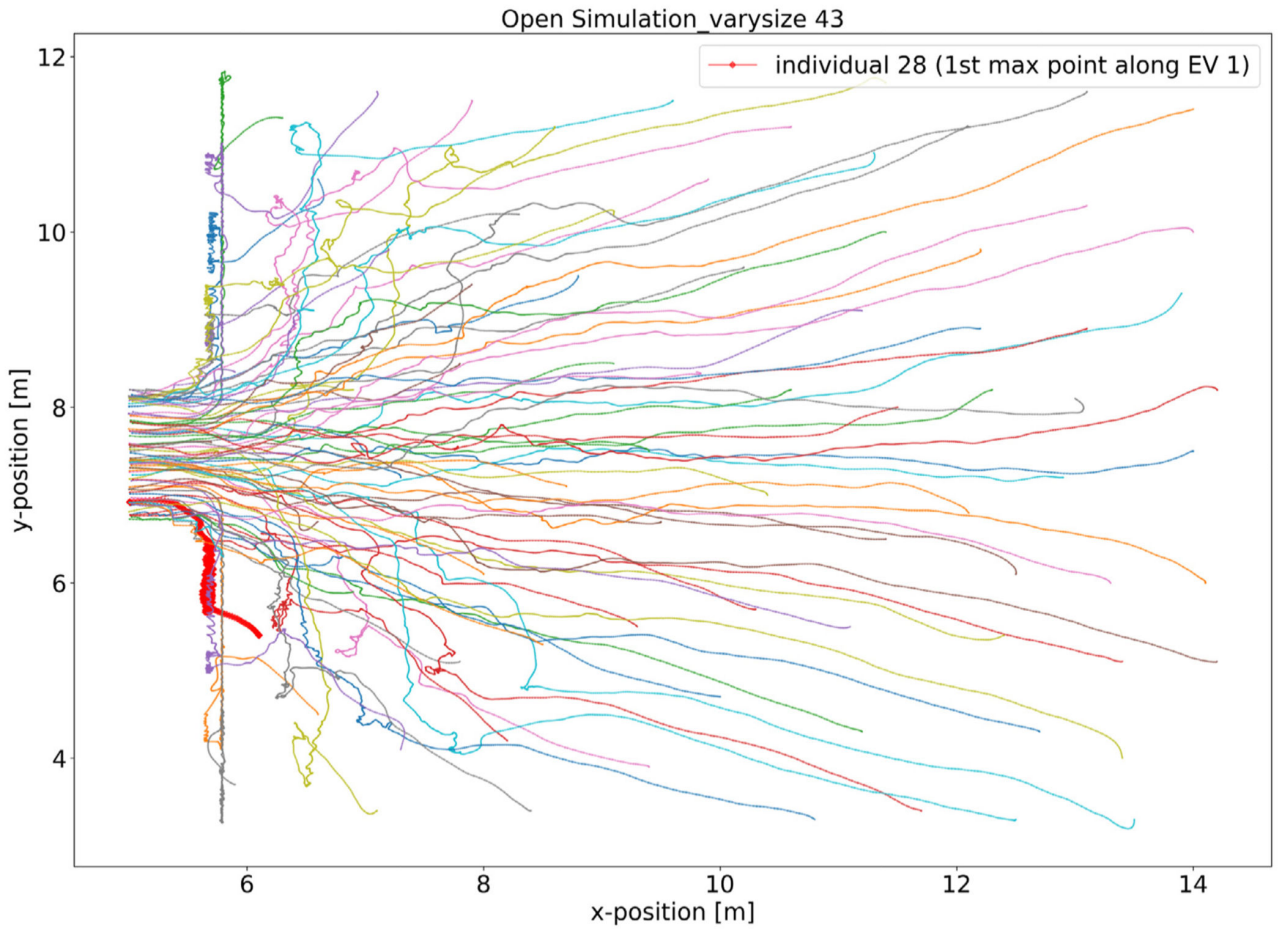
Outlier in the open system data based on the first maximum entry in the first EV. The bold red line shows the trajectory of the outlier identified by the DM with maximum entry in EV 1, which has a very short path during its run. The *x*-axis and *y*-axis show the relative size of the room (similar in the following figures). To distinguish pedestrians from the same run, we use individual numbers to label them. The label is based on the index of pedestrian in the run, which here starts from number 0. The colours of trajectories are just used to distinguish different individuals.

**Fig. 5:**
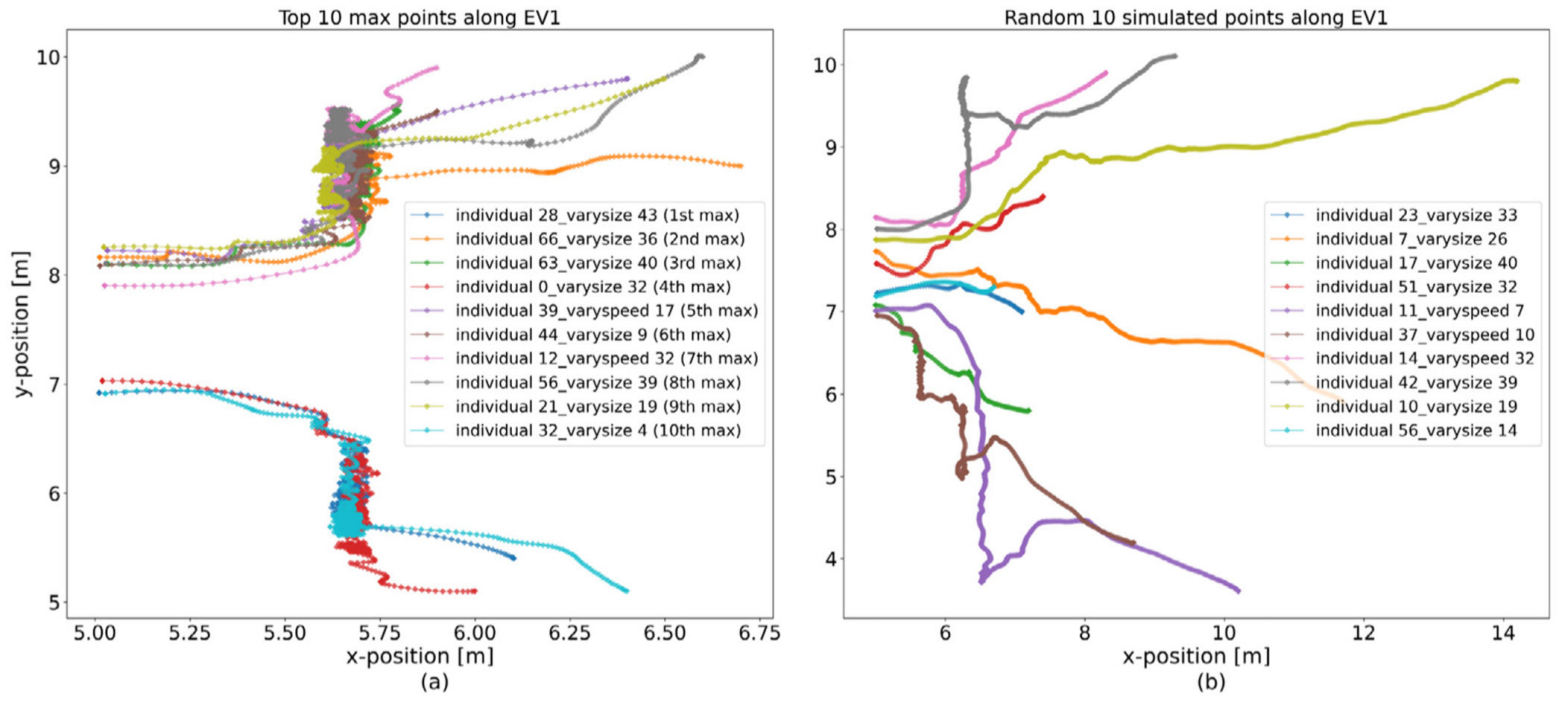
Comparison between top 10 maximum and randomly-selected data points. (a) 10 individuals’ trajectories from the top 10 maximum entries in the first EV. Each coloured line indicates a pedestrian’s trajectory in their run. Although these outliers are from different runs, we can see they have similar trajectories. In particular, these outliers all have short paths, and are roundabout while walking along the walls before they exit the room. Another interesting thing is that when these outliers exit the room, they all walk against the corridor. (b) 10 randomly-selected trajectories from all simulations of the open system. Each coloured line indicates a pedestrian’s trajectory in the run. In the legends, the parts after the individual numbers indicate the run index of the selected pedestrians, respectively. The colours of trajectories are just used to distinguish different individuals.

We consider the trajectories of pedestrians that correspond to the highest entries of the first eigenvector. Fig. 4 shows the outlier identified by the maximum entry in the context of the simulation run it emanates from. The trajectory is characterised by moving along the wall containing the bottleneck, in a direction approximately perpendicular to the predominant movement direction for some time. Fig. 5(a) shows that although they emanate from different simulation runs, the trajectories corresponding to the ten highest entries of the first eigenvector all share these characteristics. For comparison, Fig. 5(b) shows a random selection of trajectories from all simulation runs and confirms our outlier detection selects trajectories with similar characteristics that differ substantially from others in the data.

**Fig. 6:**
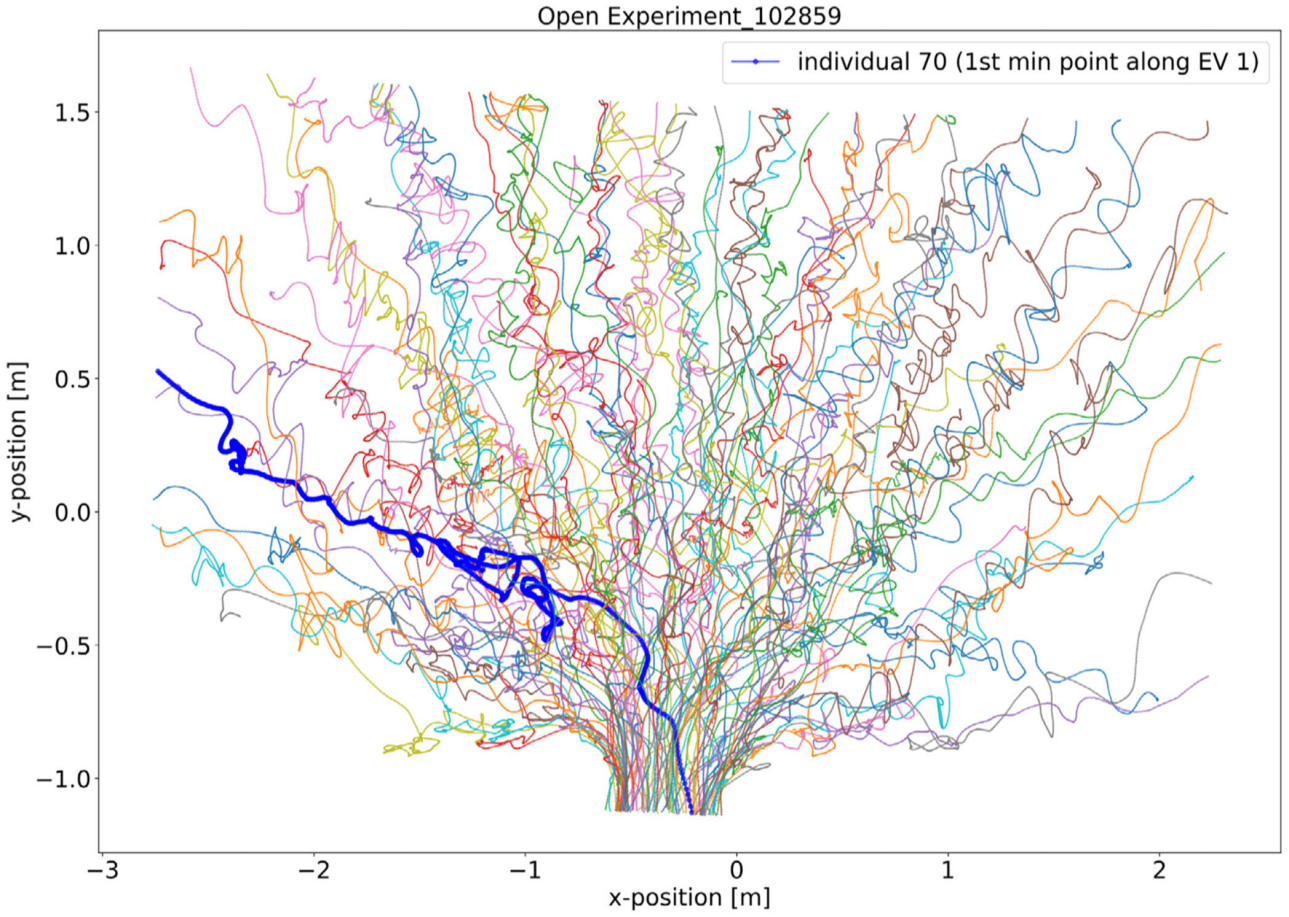
Outlier in the open system data based on the first minimum entry in the first EV. Each coloured line indicates a pedestrian’s trajectory in the run. The bold blue line shows the trajectory of the outlier identified by the DM with minimum entry in EV 1, which has a “zigzag” behaviour during the run. The colours of trajectories are just used to distinguish different individuals.

**Fig. 7:**
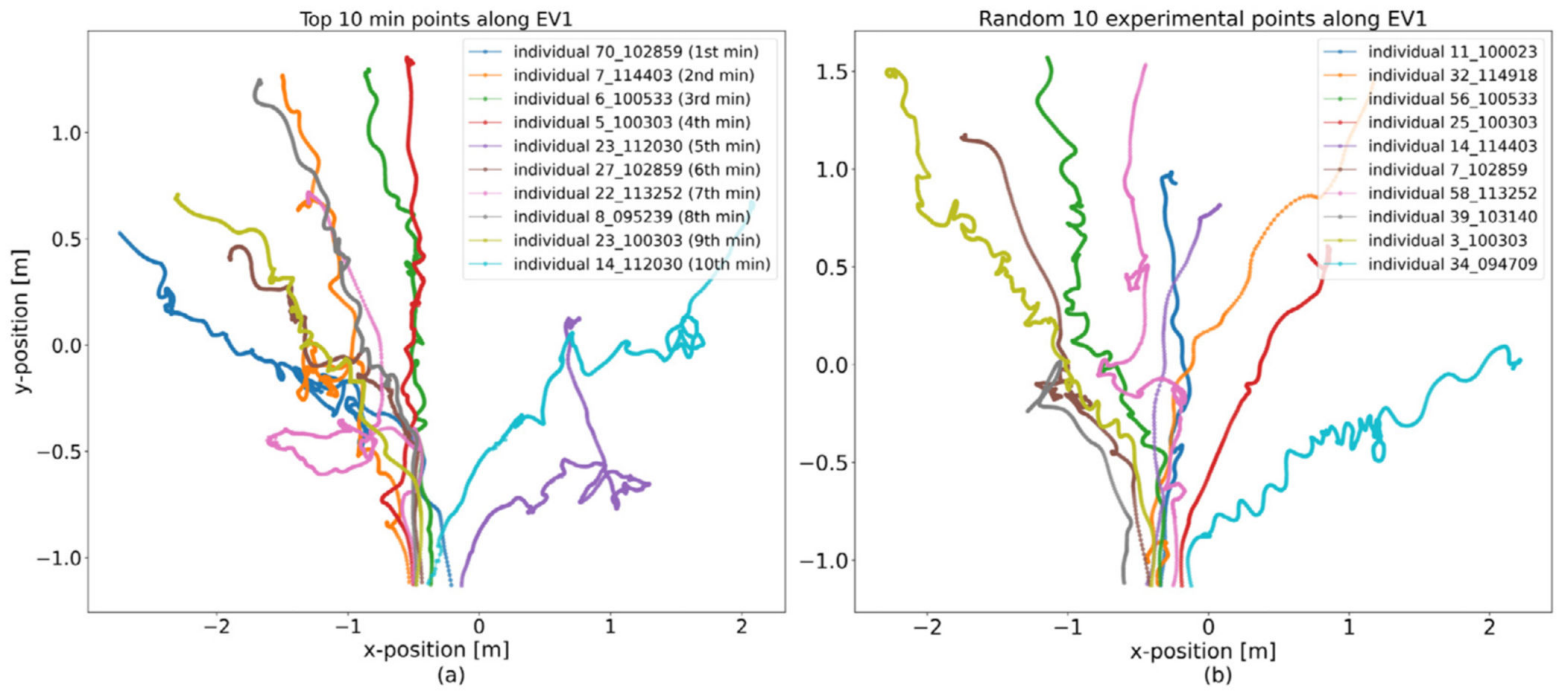
Comparison between top 10 minimum entries in EV 1 and 10 randomly-selected data points. (a) 10 individuals’ trajectories from the top 10 minimum entries in the first EV. Each coloured line indicates a pedestrian’s trajectory in their run. Although these 10 outliers are from different runs, we can see that they still have similar trajectories, such as the “zigzag” paths in the runs. Meanwhile, a few outliers’ trajectories in the centre-left even have some “parallel” paths. (b) 10 randomly-selected trajectories from all experiments of the open system. Each coloured line indicates a pedestrian’s trajectory in the run. From the *y*-axis, we can see several randomly selected individuals also have longer trajectory length than some individuals in the top 10 minimum data points. The colours of trajectories are just used to distinguish different individuals.

For comparison, we also consider the trajectories of pedestrians that correspond to the smallest entries in the first eigenvector. Fig. 6 shows the trajectory corresponding to the smallest entry in the context of the experimental run it emanates from. Compared to the outliers detected from the largest entries in the eigenvector, this outlier is less clearly characterised at first glance. It appears that the trajectory contains some backtracking, i.e., the individual returned to their previous locations several times during the march towards the door. Comparing the trajectories of the ten smallest entries in the first eigenvector to a random selection of trajectories from all experimental runs confirms this observation (Fig. 7). Notably, a few outliers with either the ten smallest or ten largest entries in the first eigenvector have some almost identical trajectories, despite emanating from different experimental or simulation runs. These results suggest that our diffusion map analysis provides a novel and straightforward way to identify outliers in pedestrian dynamics reliably.

To demonstrate outlier detection here, we arbitrarily choose ten outliers along with ten random individuals to compare. One could also choose more or less outliers to do such a comparison. In practice, one would tune a threshold to see how many outliers exist. However, this would require additional information or criteria relating to the outliers that we do not have for the data we use. We seek to demonstrate the principle of detecting outliers. Also, we here illustrate the outlier detection process by using the extreme points along the first eigenvector. There are also a few extreme points along the second eigenvector, which also show abnormal behaviours, but we do not intend to repeat a similar detection process as the first eigenvector.

## 4 Discussion

Here we show the versatility of the diffusion map in detecting patterns in large scale pedestrian trajectory data. Although we reduce the size of the data in is a pre-processing step by computing features from the raw trajectory data before implementing the diffusion map, the computation is not complicated and the number of features is small. One of the most challenging parts of implementing the diffusion map is the interpretation of its manifolds extracted from the data. In fact, the manifolds are the projections of leading variables that drive the dynamics in the original system. So, when intending to interpret manifolds, it is helpful to start by identifying variables that from the basic understanding of the dynamics of the system. For example, for pedestrian traffics, the leading variables that determine the dynamics of this system would usually be density and speed, as the fundamental diagrams indicate [38,38,39].

Regarding the interpretation of the manifolds in this work, we focus on the leading three eigenvectors. However, previous work has shown that different combinations of eigenvectors can also be useful [9,11,13]. The strengths of analysing a system via its manifolds include reducing the dimensionality of data, mapping key driving variables of the system, and finding patterns in data. In the first example, we show that the diffusion map manifold relates directly to the pedestrian density in the closed system. In the second example, we use the manifold to investigate the overlap between different data sets and identify outliers. Importantly, the diffusion map analysis is unsupervised, thus reducing the required user input, making the analysis especially suitable for an initial exploratory data investigation. The only parameter of our analysis relates to computing the similarity matrix and introduces a threshold that limits the number of neighbouring data points in the similarity matrix (via thresholding) to improve computational speed and focus on the most relevant information. Most previous works set a threshold, such that the number of neighbours in the similarity matrix is between 10 and 20 [9,11,13].

There are opportunities for further work based on our findings. For example, we focus on individual trajectories, and the data points in the manifolds thus represent individuals. It would also be interesting to explore the trajectories of groups of people or experimental runs as a whole by computing features providing information about sets of individuals. In this case, the data points in the related manifolds will indicate sets of individuals rather than individual pedestrians. Such an approach could be helpful when the goal is to investigate differences between groups, when data collection means individual trajectories are unavailable, or to keep the computations manageable when the number of individuals becomes very large. Combining the data from entire experimental runs may also make it feasible to perform unsupervised classification of data sources. In our example, it may be possible to directly distinguish between simulation and experimental run.

Another interesting extension would be to quantify the overlap between manifolds from different data sources, for example, using measures for the similarity of distributions, such as the Earth Mover’s Distance. Once quantified, the computational efficiency of the diffusion map analysis would make it a candidate for use in model calibration. According to previous research [12,40], the overall computational complexity of using diffusion maps to discover a low-dimensional manifold is 𝒪 (*N*^2^), where *N* is the number of points comprising the manifold. In our case, it will be the number of pedestrians in a data set that generates manifolds. Meanwhile, the similarity matrix **C** is relatively sparse after thresholding, which would need less storage than a full matrix in computation. The benefits of such an approach would be that the comparison between simulations and data would be performed in an unsupervised way.

In the outlier detection part, we use a qualitative (visual) way to describe the behaviours of these outliers. It might be beneficial to construct advanced quantitative methods to illustrate the outliers in the future. Also, the other extreme points along the 3-dimensional manifold can be further investigated to see if there are any other quantifiable abnormal behaviours in the data. Using outlier detection to inversely check different systems’ configurations and advance our understanding of their mechanisms would also be interesting to explore. For instance, in the manifold of the open system, some data points are away from the main cluster, which might result from the differences in the configurations of their runs.

## 5 Conclusion

In this work, we demonstrate the use of diffusion maps for unsupervised learning on pedestrian trajectories. We show that our analysis recovers patterns directly related to known driving variables in pedestrian systems. It thus presents an efficient unsupervised method for investigating patterns in high-dimensional pedestrian dynamics data. Our method can be used to directly investigate the overlap between different data sets by comparing their corresponding sub-structures in the diffusion map eigenspace. We suggest this could be useful for validating computational models for pedestrian dynamics and, if quantified, the overlap of manifolds could be used as a tool in model calibration. In addition, our diffusion map analysis can evaluate pedestrian dynamics at an individual level. We demonstrate this with the example of unsupervised outlier detection. In summary, we suggest diffusion map presents a useful complementary tool for data exploration and analysis in pedestrian dynamics.

## Supplementary Material

Supplementary material related to this article can be found online at https://doi.org/10.1016/j.physa.2023.129449.

Supplemental data

## Figures and Tables

**Table 1:** Descriptive statistics of the 5 analysed data sets.

Data set	No. of trajectories	Travel distance (mean ± s.d.) [m]	Min. travel distance [m]	Max. travel distance [m]	Travel time steps (mean ± s.d.)	Min. travel time steps	Max. travel time steps
Closed experiment	994	93.73 ± 45.61	30.65	230.27	4960.73 ± 1157.51	2260	7678
Open experiment	3231	4.37 ± 2.04	0.01	12.16	829.91 ± 452.01	3	2078
Open simulation 1	4000	15.35 ± 5.94	3.56	36.73	4312.02 ± 453.33	3475	5417
Open simulation 2	4000	6.75 ± 2.61	0.47	12.3	1083.7 ± 26.21	994	1134
Open simulation 3	4000	6.73 ± 2.61	0.49	12.06	1005.26 ± 20.03	960	1048

**Table 2:** Features used to describe the pedestrian dynamics data. The angles in the table are all in radians. We compute a pedestrian’s nearest neighbours for every time step during their run.

No.	Observable	Interpretation
(1)	$\bar{h_{i1}(t)}$	Mean of aheadnesses between *i* and its first nearest neighbours
(2)	$\hat{h_{i1}(t)}$	Variance of aheadnesses between *i* and its first nearest neighbours
(3)	$\bar{l_{i1}(t)}$	Mean of the leftnesses between *i* and its first nearest neighbours
(4)	$\hat{l_{i1}(t)}$	Variance of the leftnesses between *i* and its first nearest neighbours
(5)	$\bar{d_{i1}(t)}$	Mean of distances to an individual’s first nearest neighbours
(6)	$\bar{\gamma_{i1}(t)}$	Mean of angles of directions between *i* and its first nearest neighbours
(7)	$\bar{h_{i2}(t)}$	(7)–(12) are similar to (1)–(6) while use the second nearest neighbours
(8)	$\hat{h_{i2}(t)}$	
(9)	$\bar{l_{i2}(t)}$	
(10)	$\hat{l_{i2}(t)}$	
(11)	$\bar{d_{i2}(t)}$	
(12)	$\bar{\gamma_{i2}(t)}$	
(13)	$\bar{h_{i3}(t)}$	(13)–(18) are similar to (1)–(6) while use the third nearest neighbours
(14)	$\hat{h_{i3}(t)}$	
(15)	$\bar{l_{i3}(t)}$	
(16)	$\hat{l_{i3}(t)}$	
(17)	$\bar{d_{i3}(t)}$	
(18)	$\bar{\gamma_{i3}(t)}$	
(19)	$\bar{\alpha_{i}^{1}(t)}$	Mean of individual’s angle difference in every 1 time step
(20)	$\hat{\alpha_{i}^{1}(t)}$	Variance of individual’s angle difference in every 1 time step
(21)	$\bar{\alpha_{i}^{5}(t)}$	Mean of individual’s angle difference in every 5 time steps
(22)	$\hat{\alpha_{i}^{5}(t)}$	Variance of individual’s angle difference in every 5 time steps
(23)	$\bar{\alpha_{i}^{10}(t)}$	Mean of individual’s angle difference in every 10 time steps
(24)	$\hat{\alpha_{i}^{10}(t)}$	Variance of individual’s angle difference in every 10 time steps
(25)	$\bar{\alpha_{i}^{20}(t)}$	Mean of individual’s angle difference in every 20 time steps
(26)	$\hat{\alpha_{i}^{20}(t)}$	Variance of individual’s angle difference in every 20 time steps
(27)	$\bar{s_{i}^{20}(t)}$	Mean of individual’s travel distances in every 20 time steps

## Data Availability

The experimental data used in this paper is publicly accessible, as described in the citations. While the simulated data will be made available on request, it is reproducible following the detailed description and references in the paper. The code for the essential steps of the diffusion map analysis is supplied in the supplementary material.
